# Supplementary material for: Identification of photoperiod-induced specific miRNAs in the adrenal glands of Sunite sheep (Ovis aries)
Source: Front Vet Sci. 2022 Jul 22;9:888207. doi: 10.3389/fvets.2022.888207 (PMC9354845; doi:10.3389/fvets.2022.888207)
Supplement: Supplementary file 1 [file Data_Sheet_1.ZIP › Supplementary file/miRDeep2 file.zip!/miRDeep2 file/Novel_Structure/SP42A1/Novel_358.pdf]

Provisional ID	:	NC_019458.2_1005
Score total	:	2.4
Score for star read(s)	:	-1.3
Score for read counts	:	0
Score for mfe	:	2.1
Score for randfold	:	1.6
Score for cons. seed	:	
Total read count	:	76
Mature read count	:	60
Loop read count	:	0
Star read count	:	16

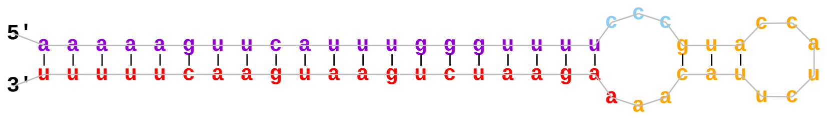

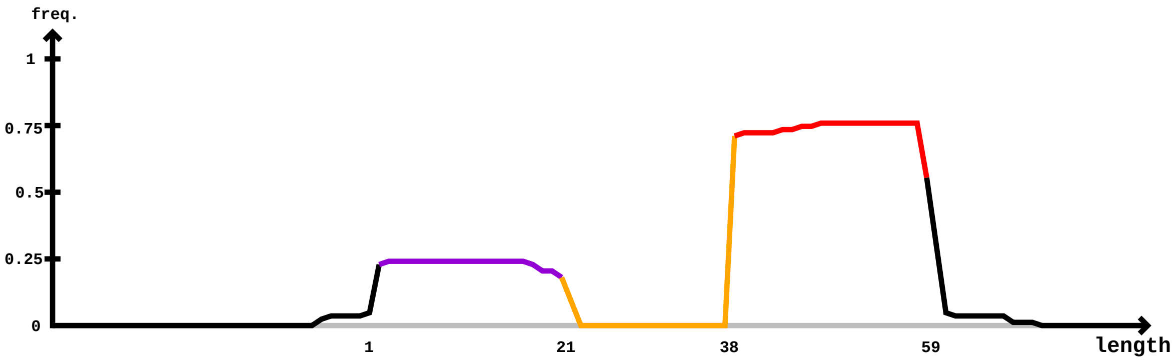

Star   Mature

```
5'- caaaaugaaugaacuaugauauugcguuggccaaaaaguucauuuggguuuucccguaccaucuuacaaaagaaucugaaugaacuuuuuggccaacccaauacauucauca   -3'   obs
    caaaaugaaugaacuaugauauugcguuggccaaaaaguucauuuggguuuucccguaccaucuuacaaaagaaucugaaugaacuuuuuggccaacccaauacauucauca         exp
    ....(((((((........(((((.(((((((((((((((((((((((((((...(((......)))...))))))))))))))))))))))))))).))))))))))))..   reads   mm   sample
    ..........................uuggccaaaaaguucGuuuggg................................................................   1       1    seq
    ..........................uuggccaaaaagCucauuugggu...............................................................   1       1    seq
    ...........................uggccaGaaaguucauuugggu...............................................................   1       1    seq
    ...............................caaaaaguucGuuuggguuuuc...........................................................   1       1    seq
    ................................aaaaaguucGuuuggguuu.............................................................   2       1    seq
    ................................aaaaGguucauuuggguuuu............................................................   1       1    seq
    ................................aaaaaguucGuuuggguuuu............................................................   12      1    seq
    .................................aaaGguucauuuggguuuu............................................................   1       1    seq
    .....................................................................aaAaaucugaaugaacuuuu.......................   17      1    seq
    .....................................................................aaAaaucugaaugaacuuuuu......................   42      1    seq
    ......................................................................agaaucugaaugaacuuuuugg....................   1       0    seq
    ..........................................................................ucugaaugaacuuuuuggccaacA..............   1       1    seq
    ............................................................................ugaaugaacuuuuuggccCacc..............   1       1    seq
    ..............................................................................aaugCacuuuuuggccaacccaa...........   1       1    seq
```
